# Experimental Investigation on the Transpiration Cooling Characteristics of Sintered Wire Mesh in Plain Weave

**DOI:** 10.3390/mi13030450

**Published:** 2022-03-16

**Authors:** Yubo Peng, Guoqiang Xu, Xiang Luo, Jian He, Dongdong Liu

**Affiliations:** National Key Laboratory of Science and Technology on Aero Engines Aero-Thermodynamics, The Collaborative Innovation Center for Advanced Aero-Engine of China, Beihang University, Beijing 100191, China; dream070141@buaa.edu.cn (Y.P.); 04822@buaa.edu.cn (G.X.); clhejian@126.com (J.H.)

**Keywords:** sintered metal wire mesh, transpiration cooling, heat transfer, porous material

## Abstract

We experimentally investigate the transpiration cooling characteristics of a porous material, sintered wire mesh. Three samples with different porosities in a plain weave structure are tested with various blowing ratios in an open-loop wind tunnel with a heated mainstream flow. The temperature on the surface of the porous material is measured by an infrared camera to obtain the cooling efficiency. The measurements reveal nonuniform distributions of the surface temperature and the cooling efficiency in both the flow direction and the transverse direction. The averaged cooling efficiency on the surface first decreases and then increases with the blowing ratio, but increases and then decreases with the porosity of the material. The internal cooling by forced convection and its combination with the external film cooling from the transpiration cooling are considered to be attributed to those two cooling characteristics, respectively. Finally, we propose a modified blowing ratio to collapse the minima of the blowing ratio for all tested samples, providing an universal transition for the decreasing and increasing branches for all tested samples in the relation between averaged cooling efficiency and blowing ratio.

## 1. Introduction

Conventional cooling technologies such as film cooling and impingement cooling, although providing thermal protection of high-temperature components of aeroengine, have almost reached their performance limits. This not only makes improvement of their cooling performance much more difficult, but also becomes an obstacle in the ever-increasing demand for aeroengine efficiency.

Transpiration cooling uses porous material as a cooling wall which has a small pore size and extremely dense pore distribution with a typical size scale of 100 μm, inducing internal forced convection inside the material and external film cooling on the surface. The former reduces the overall average temperature of the solid framework of the porous material, while the latter generates a continuous and relatively uniform coolant film with low temperature on the surface of the porous material. This so-called “air membrane” is capable of separating the surface from the high temperature mainstream flow, inducing a further decrease in the surface temperature and, correspondingly, the improvement of cooling efficiency. Therefore, transpiration cooling has the highest theoretical cooling efficiency [1] and has shown its importance in various applications [2,3,4,5].

For transpiration cooling, several parameters—including the flow condition, the porous material and its geometric parameter—are deemed vital in determining the cooling characteristics. For the flow condition, one commonly investigated parameter is the blowing ratio, which measures the ratio of the mass flow rate between the coolant and the heated mainstream flow. The research of Jiang et al. [6] showed, that under the condition of constant thermal-physical properties, the increase in transpiration cooling blowing ratio greatly reduces the wall temperature and the convective heat transfer coefficient between the wall and the main stream. For a blowing ratio of 1%, the convection heat transfer coefficient was reduced by about 50%. Langener et al. [7] explored the transpiration cooling efficiency of C/C materials under the condition that the mainstream is subsonic or supersonic. The experiment revealed that the mainstream condition has little effect on the transpiration cooling efficiency in the current conditions. Subsequently, they selected air, argon and helium as the cooling medium for transpiration cooling. The experiments indicated that the specific heat capacity and blowing ratio of the cooling medium were the most important factors affecting transpiration cooling efficiency [3].

For the matrix porous material used for transpiration cooling, a variety of materials can be used as the matrix for transpiration cooling. Transpiration cooling based on sintered metal powder has been designed and applied in the nose cone of some hypersonic vehicles or scramjet combustors [3,8]. The porous materials synthesized using a ceramic matrix such as carbon/carbon has been applied for transpiration cooling in rocket engine thrust chamber cooling [9,10,11,12]. In recent years, sintered wire mesh has also been utilized as the liquid fuel injection panel of rocket engine thrust chamber [13] for transpiration cooling research. Some researchers also applied sintered wire mesh on the turbine blade wall of an aeroengine and preliminary explored the application possibility of turbine blade transpiration cooling [14,15,16].

To design and apply the transpiration cooling in practical applications, a full understanding of the structure parameters of the porous material for the transpiration cooling is required. Sintered metal powder, sintered wire mesh and C/C composites are several popular transpiration cooling matrix materials. The transpiration cooling structure of sintered metal powder has been widely studied. Liu et al. [17] investigated the effect of metal powder particle size on transpiration cooling efficiency. The cooling performance of the porous material with the smaller particle diameters was found to have better performance, mainly due to the enhanced convection heat transfer inside the material. Gan Huang et al. [18] studied the effect of liquid phase transition on the transpiration cooling of sintered metal powder. The results illustrated that the average cooling efficiency and the maximum wall temperature increased with the decrease in metal powder size from 600 μm to 90 μm. Langener et al. [19] explored the effect of material thickness and porosity on the cooling efficiency of composite carbon/carbon materials (C/C). It was found that material thickness and porosity do not affect cooling efficiency. Min et al. [20] experimentally studied a variety of transpiration cooling materials fabricated by 3D printing. The experiments demonstrated that—among the three geometric parameters of transpiration cooling structure, such as porosity, material internal fluid solid contact area and cold air outlet area—the material internal fluid solid contact area has the greatest impact on transpiration cooling efficiency.

Sintered wire mesh uses metal wires as the raw material. The metal wires are woven into a screen in a specific way, usually in the form of a Dutch or plain weave, and then the multi-layer wire mesh is reasonably arranged and manufactured via the sintering process. Sintered metal wire mesh porous material has good controllability: firstly, when weaving the wire mesh, the weaving method of different layers of wire mesh can be changed to adjust its mechanical properties to form a fiber-reinforced anisotropic material that can be adapted to loads in different directions; secondly, during the rolling process, the wire mesh can be deformed by varying the applied load, thereby changing the pore size, and then the porosity and permeability of the wire mesh [21].

Recently, Xu et al. [22] explored the transpiration cooling efficiency of sintered wire mesh in Dutch weave form with three kinds of porosity. The results showed that the cooling efficiency did not increase with the porosity. Ma et al. [23] studied the multi-layer structure of sintered wire mesh with different porosity combinations in the thickness direction. The experiment results indicated that the divergent cooling efficiency increased with the average porosity in the thickness direction of sintered wire mesh, and adjusting the order of wire mesh layers with different porosities will affect the transpiration cooling efficiency. However, for the sintered wire mesh in plain weave, which is another important weaving form for sintered wire mesh, its transpiration cooling characteristics have not been fully elucidated.

In this study, the sintered wire mech in plain weave is used as the matrix material for transpiration cooling. An open-loop wind tunnel with a heated mainstream flow is applied for experimental measurement. The surface temperature on three test samples with different porosity are captured by infrared camera to obtain higher cooling efficiency at various blowing ratios. The presented relation between the transpiration cooling efficiency and blowing ratio or porosity is capable of providing further insights in the transpiration cooling of sintered wire mesh for practical applications.

## 2. Materials and Methods

### 2.1. Sintered Wire Meshes

**Figure 1:**
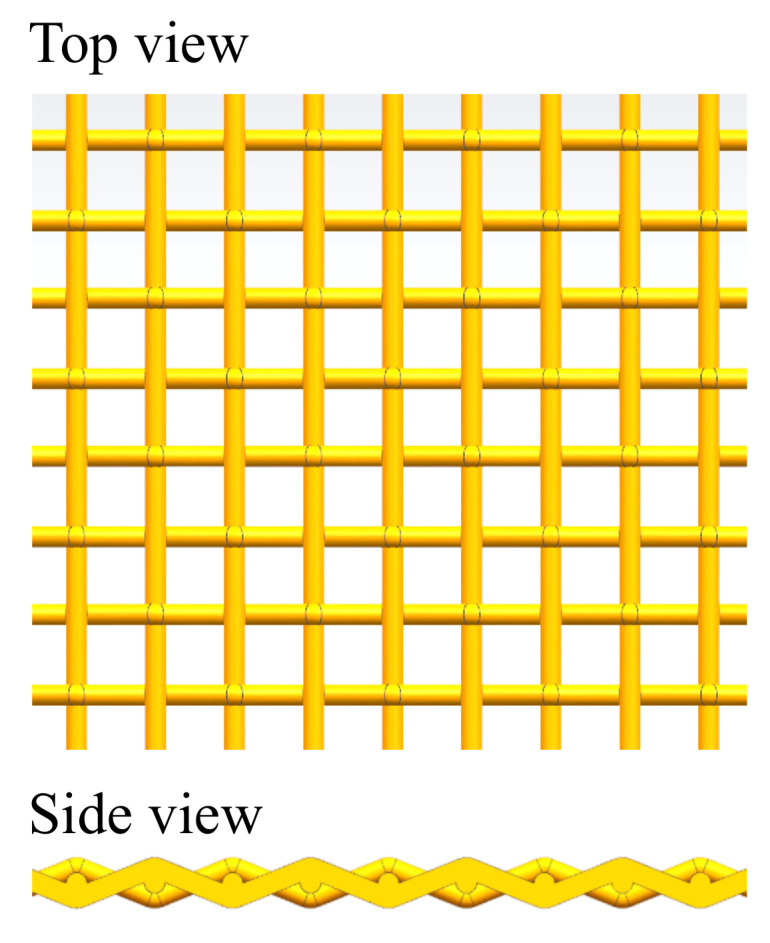
Schematic of the wire mesh structure. The diameter of the wire is 140 μm.

Sintered metal wire mesh is manufactured using stainless steel wires with the diameter of 140 μm. The manufacture process consists of two steps: Firstly, the wires are woven to produce a single-layer structure with uniform pattern, as shown in Figure 1. The weaving method of the sintered wire mesh used in this study is plain weave, one of the most commonly used weaving methods. Subsequently, several single-layer woven wire meshes are exposed sequentially to compression, rolling and vacuum sintering, to produce the ultimate sintered wire mesh with multiple-layer structure. For all the tested sample, the dimensions of the porous material is kept at 30 mm in width, 90 mm in length and 6 mm in thickness.

To obtain test samples with different porosity *ε*, i.e., the void fraction in the volume of the matrix, the quantity of the layers was changed from 40 to 54 in the manufacturing process. Typically, by increasing the layer quantity of the sintered wire meshed and compressing the matrix into a same size mode, the porosity of the manufactured sample decreases. The geometric detail of the sample is shown in Table 1.

By compressing and sintering several layers of wire meshes into a porous material, the geometry deforms dramatically. For the unsintered wire mesh, the gap between the stainless steel wire is around 500 μm. For the sintered wire mesh, the average pore diameter changes from 165 μm to 71.8 μm by increasing the quantity of sintered layer from 40 to 54. Correspondingly, the porosity of the test sample varies from 56.5% to 38.9%. Therefore, as shown in Table 1, although diameter of the stainless steel wire does not determine the size of the pore, it preliminary sets the possible scale of the pore size in the sintered wire mesh.

### 2.2. Experimental Apparatus

**Figure 2:**
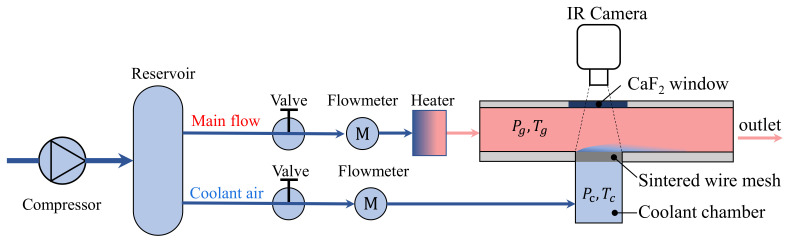
Schematic of the experiment apparatus for the measurement of the transpiration cooling characteristics of the sintered wire mesh.

The transpiration cooling experiment is conducted in a low-speed open-loop wind tunnel, as shown in Figure 2. The air flow of the experiment is provided by a compressor connected to an air reservoir. Two air flows extracted from the reservoir are controlled by two gas control valves separately. At each air flow channel, one FCI-ST98 thermal flowmeter accurately measures the flow rate with the uncertainty of ±0.5%. After the flowmeter, the air in the mainstream is heated by an electric heater to set temperature $T_{g}$ ranging from 90 °C to 120 °C before it purges into the rectangular channel of test section, which is 30 mm wide and 45 mm high. For the cooling air, it is led into a coolant chamber for pressure stabilization, in which its temperature $T_{c}$ and pressure $P_{c}$ are measured. The transpiration cooling test piece, the sintered wire mesh, is assembled between the coolant chamber and mainstream channel, allowing the cooling air flow through this porous material and generating the transpiration cooling.

Two calibrated K type thermocouples are fixed in the flow channels to measure the temperature of the coolant and hot air in the mainstream. To capture the two dimensional temperature distribution on the sintered wire mesh, an infrared camera (model: FLIR sc7700m) with focus on the surface of the test sample is set opposite the sample with a CaF${}_{2}$ window as the observation window. It is a short wave infrared system with a receiving wavelength range between 2.5 μm and 5 μm and a measurement range between −20 °C and 1500 °C. Before the experiment, the camera was calibrated by a K type thermocouple to reach an uncertainty of $\pm 0.5^{{}^{\circ}}$C. The resolution of the infrared measurement is 640 pixels × 512 pixels.

### 2.3. Data Processing

In the research on transpiration cooling, the blowing ratio *F* is commonly used to measure the mass flow ratio between the cooling air and hot air in the mainstream. The expression of *F* can be defined as following

(1) $$F=\frac{\rho_{c}u_{c}}{\rho_{g}u_{g}},$$

where $\rho_{c}$ and $\rho_{g}$ are the densities of the coolant and main flow, respectively, and $u_{c}$ and $u_{g}$, respectively, the velocities of the coolant and main flow. However, it is difficult to accurately measure the density and flow velocity of the flow, especially for the coolant. Therefore, we use the blowing ratio according to the following formula

(2) $$F=\frac{\dot{m_{c}}/A_{c}}{\dot{m_{g}}/A_{g}}=\frac{\dot{m_{c}}A_{g}}{\dot{m_{g}}A_{c}},$$

where $\dot{m_{c}}$ and $\dot{m_{g}}$ are, respectively, the mass flow rates of the coolant and the main flow, and $A_{c}$ and $A_{g}$, respectively, the flow area of the coolant channel and mainstream channel. Since the mass flow rate is measured by the thermal flowmeter, the uncertainty of the calculated blowing ratio *F* is very low. It has to be noted that the blowing ratio *F* defined in this study does not contain the geometric parameter of the test sample.

The transpiration cooling efficiency, which measures the cooling effectiveness, is defined as following

(3) $$\eta =\frac{T_{wa}-T_{w}}{T_{wa}-T_{c}},$$

where $T_{wa}$ is the surface temperature of the test sample in the case of F=0, and $T_{w}$ the surface temperature of the test piece when F≠0.

## 3. Results and Discussion

### 3.1. The Effect of Blowing Ratio and Porosity on the Surface Temperature Distribution

**Figure 3:**
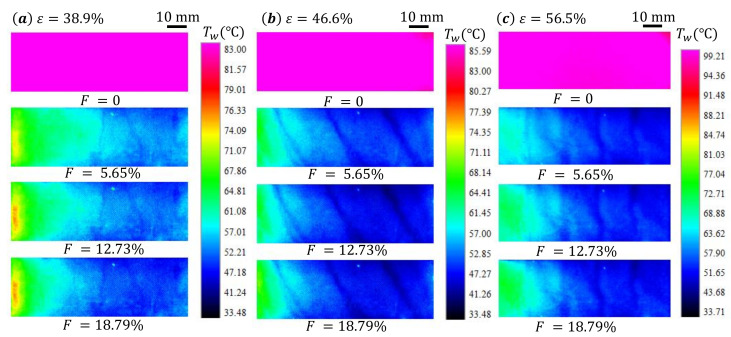
Temperature contour on the surface of the sintered wire mesh for different porosity and blowing ratios. The heated mainstream air flows from left to right, which is denoted as the *x*-axis. The legends are adjusted to show the distribution of the temperature on the surface when F≠0.

When cooling air flows through the sintered wire mesh, it cools the surface by forced convection inside the medium and forming a air film between the hot air and the surface. Therefore, the temperature on the surface of the sintered wire mesh is much lower than that when F=0. It has to be noted that the surface temperature $T_{wa}$ on the surface of the sintered wire mesh is mostly uniform when F=0. We measure the $T_{wa}$ to calculate the cooling efficiency when F>0: for ε=38.9%, $T_{wa}=98.2^{{}^{\circ}}$C; for ε=46.6%, $T_{wa}=92.4^{{}^{\circ}}$C; for ε=56.5%, $T_{wa}=99.1^{{}^{\circ}}$C. When F>0, the surface temperature $T_{w}$ significantly reduces under the cooling effect of the cooling air (as shown in Figure 3a).

We observe that the temperature distribution on the surface of the sintered wire mesh becomes non-uniform. Typically, the surface temperature decreases along the flow direction, denoted as *x*-axis, in the mainstream. We also note the variation of surface temperature in the transverse direction, denoted as *y*-axis. The variation of temperature in *x* direction can be caused by either the decreasing of pressure along the mainstream or the entrance effect of the heat transfer. The former essentially provides a decreasing outlet pressure for the cooling flow through the porous material, inducing redistribution of the mass flow through the porous medium. Since the downstream has a lower pressure than the upstream, larger pressure drop in the downstream results in more cooling air flowing out in the downstream of the porous medium, leading to better cooling in that area. The latter is related to the increase in boundary layer thickness with the accumulating of the cooling air along the flowing direction, resulting in a relatively higher heat transfer efficiency in the upper part and a lower one in the lower part. For the uneven temperature distribution in the *y* direction, it can be generated by the boundary lay flow in the corners of the mainstream channel. Generally speaking, low velocity flow in the corner area reduces the heating effect of the hot air in the mainstream and generates low surface temperature near the corner areas.

With the increase in the blowing ratio *F* from 5.65% to 18.79%, the temperature on the surface of the sintered wire mesh decreases, suggesting the enhancing of the cooling effect by the cooling air. This phenomena are all observed on the surface of sintered wire mesh with different porosity *ε*, as shown in Figure 3.

**Figure 4:**
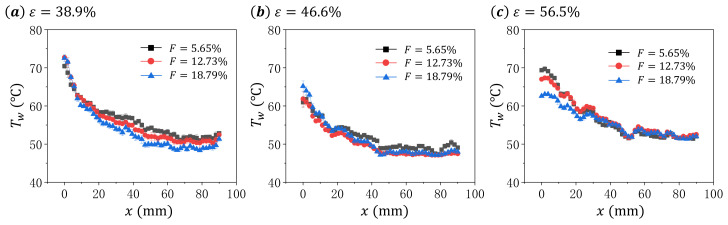
The distribution of surface temperature $T_{w}$ along the central line of the test sample for different blowing ratio *F* at same porosity *ε*: (**a**) ε=38.9%, (**b**) ε=46.6%, and (**c**) ε=56.5%.

**Figure 5:**
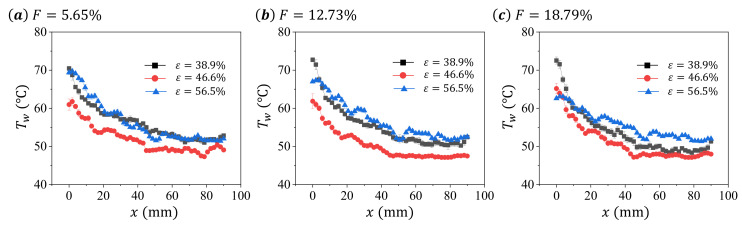
The distribution of surface temperature $T_{w}$ along the central line of the test sample for different porosity *ε* at same blowing ratio *F*: (**a**) F=5.65%, (**b**) F=12.73%, and (**c**) F=18.79%.

To better understand the distribution of the surface temperature $T_{w}$ on the test sample, we extract the $T_{w}$ on the central line along *x* direction for each cases shown in Figure 3, and present the results in Figure 4 and Figure 5. The data are arranged differently in these two figures to better illustrate the effects of blowing ratio *F* and porosity *ε* on the surface temperature $T_{w}$ distribution of the sintered wire mesh.

As shown in Figure 4, the surface temperature $T_{w}$ generally decreases with *x*, and some fluctuations of $T_{w}$ along the distribution. This may be caused by the rough surface of the sintered wire mesh generated during the manufacture. With the increase in blowing ratio *F*, we find different changing patterns for the three test samples. For the material with ε=38.9%, the increase in *F* causes insignificant variation of $T_{w}$ in the upper part of test sample (around x<10 mm), but significant decreasing of $T_{w}$ in the lower part of test sample (around x>10 mm). For the sample with ε=46.6%, the increase in *F* induces the temperature decrease in all the surface of the test sample. For the sample with ε=56.5%, we observe the decrease in $T_{w}$ in the upper part of the sample (around x<20 mm) with the increase in *F*. Therefore, the transpiration cooling characteristics of the sintered wire mesh change with the porosity.

It must be noted that for sintered wire mesh, its internal heat transfer characteristic generally increases with the mass flow rate of the cooling air [24]. When applying sintered wire mesh for transpiration cooling, the cooling characteristics are co-determined by the internal cooling and the external film cooling which have yet be fully understood. However, we can infer from Figure 4 that the cooling characteristics of the sintered wire mesh significantly depends on the porosity of the material.

In Figure 5, we compare the surface temperature $T_{w}$ distribution for different test samples at same *F*. Similar distribution trends of $T_{w}$, decreasing with *x*, can be found among different samples in all presented *F*. Although we observe the difference in $T_{w}$ among the samples with same *ε*, it has to be noted that the value of $T_{wa}$ differs between samples. Therefore, simply comparing the surface temperature among samples with the same *ε* may be meaningless.

### 3.2. The Effects of Blowing Ratio and Porosity on the Cooling Efficiency

**Figure 6:**
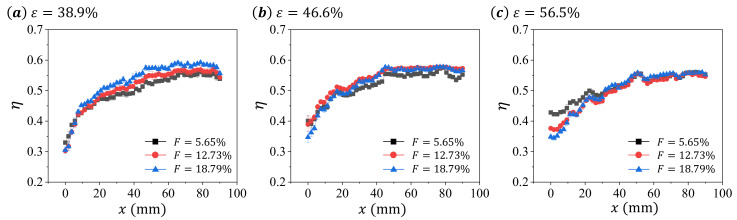
The distribution of cooling efficiency *η* on the central line of the test sample for different blowing ratio *F* at same porosity *ε*: (**a**) ε=38.9%, (**b**) ε=46.6%, and (**c**) ε=56.5%.

**Figure 7:**
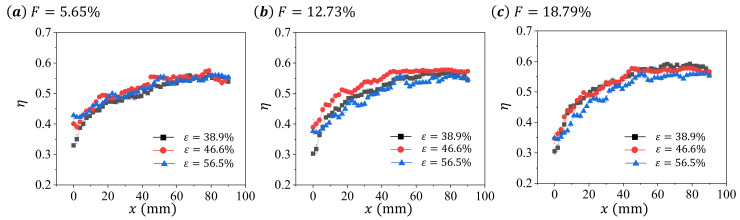
The distribution of cooling efficiency *η* on the central line of the test sample for different porosity *ε* at same blowing ratio *F*: (**a**) F=5.65%, (**b**) F=12.73%, and (**c**) F=18.79%.

To demonstrate the effects of blowing ratio *F* and porosity *ε* on the cooling efficiency *η* of the test samples, we calculate the cooling efficiency *η* from surface temperature based on Equation (3) and present the result in Figure 6 and Figure 7.

Since high surface temperature results in low cooling efficiency, the changing characteristics of *η* is inverse to that of $T_{w}$. For *η*, this increases with *x* first and then becomes saturated for all presented data in Figure 6 and Figure 7. We note that the transition position between these two trends are all around x≈50 mm for all presented data. For the non-uniform distribution of *η*, this can be caused by either the decrease pressure in the mainstream channel or the entrance effect of the heat transfer. Since the pressure drop for the air flowing through the porous media varies between samples [24], the effect of pressure decrease on *η* may be different among samples. Therefore, we speculate that the entrance effect may play an more important role in the determining of the distribution of *η*, since the mass flow rate of the cooling air is generally low (less than 1%).

For the transpiration cooling at same *F* among test samples, we observe insignificant variation at low *F* (F=5.65%), and significant differences at large *F*. In addition, the sample with ε=46.6% has a higher *η* than other samples in all *F* (Figure 7), suggesting a non-monotonic changing characteristic between the cooling efficiency of the sample and its porosity.

### 3.3. The Effect of Blowing Ratio and Porosity on the Averaged Cooling Efficiency

**Figure 8:**
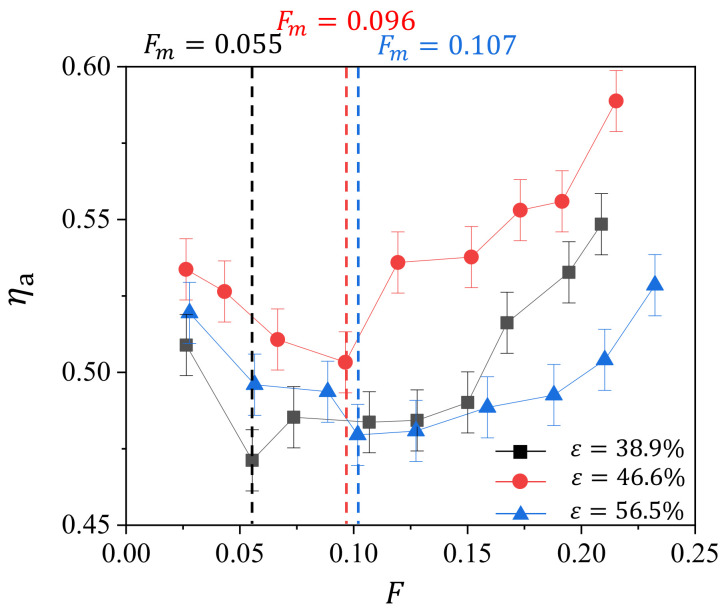
Variation of average cooling efficiency $\eta_{a}$ with blowing ratio *F* for different porosity *ε*.

To further compare the cooling efficiency among the samples at all tested blowing ratios, we calculate the averaged cooling efficiency $\eta_{a}$ by averaging the cooling efficiency on the surface of the sample for one case. We plot the dependence of $\eta_{a}$ with *F* for the three tested samples and present the result in Figure 8.

Generally, we observe that, for all the samples, $\eta_{a}$ first decreases and then increases with *F*, giving minima of $\eta_{a}$ in the curves of transpiration cooling characteristics. We highlight the values of $F_{m}$ at the minima of $\eta_{a}$ for all the samples, and find the monotonic relation between $F_{m}$ and *ε*. For this transpiration cooling characteristics of sintered wire mesh, it resembles the characteristics of the film cooling on the endwall of gas turbine vane with full coverage of film cooling holes [25]. The film cooling efficiency on the endwall first decreases and then increases with the blowing ratio. This can be explained by the following: when blowing ratio is small, i.e., the velocity of the coolant is low, the cooling air easily enters into the low velocity region of the boundary layer in the mainstream channel, giving full coverage of the cooling air film on the endwall; with the increase in blowing ratio, the cooling air becomes difficulty to enter the boundary layer, giving rise to the decrease in cooling efficiency; with the further increasing of the blowing ratio, the cooling air with high velocity enhances the heat transfer on the surface, resulting in the increase in cooling efficiency. Therefore, the cooling efficiency decreases and then increases with the blowing ratio.

We speculate that this reasoning can also be applied in the transpiration cooling of the sintered wire mesh. Although the transpiration cooling consists of the internal cooling and external cooling, the internal cooling generally increases with the mass flow rate of the cooling air. Therefore, these transpiration cooling characteristics are mostly determined from the external cooling of the sintered wire mesh, which is essentially the film cooling. However, we still has to admit that both the internal cooling and external cooling determine the transpiration cooling of the sintered wire mesh.

Among the three samples, $\eta_{a}$ of the sample with ε=46.6% overwhelms that of the other samples in all tested *F*. Since the internal cooling monotonically increases with the decrease in porosity, this phenomenon can be explained from the side of external cooling. Smaller porosity causes enhanced heat transfer in the internal cooling, but the higher velocity and larger velocity difference in the cooling air, caused by the smaller size of the pore, may induce more intense mixing between the cooling air and mainstream flow and correspondingly reducing of the cooling efficiency. In addition, a large pore diameter may cause the decrease in the pore density on the surface, reducing the effect of external cooling. Therefore, the sample with ε=46.6% has the highest cooling efficiency among the three tested samples. For the crossing of the heat transfer characteristic curves for the samples with ε=38.9% and ε=56.5%, it can be the enhanced heat transfer of the internal cooling at high *F*.

**Figure 9:**
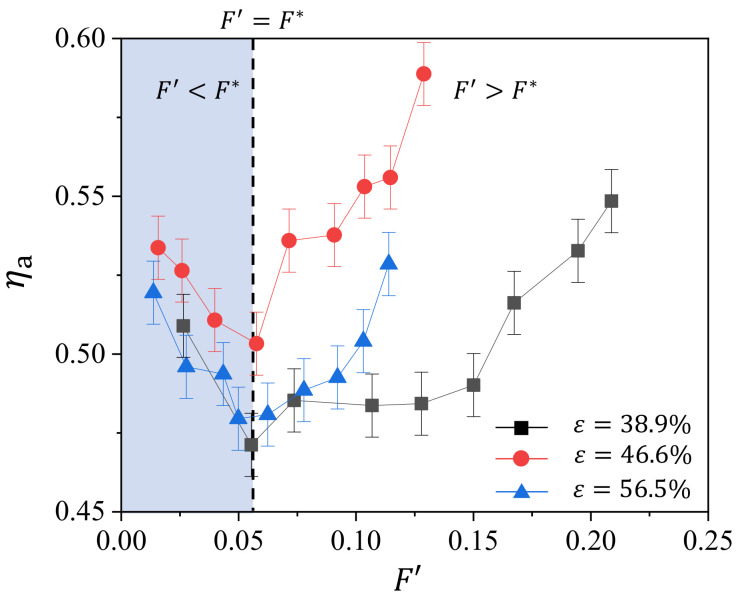
Variation of average cooling efficiency $\eta_{a}$ with the modified blowing ratio $F^{\prime }$ for different porosity *ε*.

To obtain further insights into the effect of geometry parameter on the transpiration cooling efficiency of the sintered wire mesh, we try to provide a modified blowing ratio $F^{\prime }$ that includes the factor of material’s geometric parameter. For the blowing ratio used in this study, it does not consider the geometry factors of the porous medium, e.g., the porosity and the pore size. We can use the porosity to roughly estimate the flow area, which may give a better estimation of the cooling air velocity, or we can simply use the pore diameter, which essentially is the diameter of the hole in film cooling and determines the density of the hole on the surface. For the sake of convenience, we apply the normalized average pore diameter $d/d_{1}$ (as shown in Table 1) for the modified blowing ratio $F^{\prime }=Fd/d_{1}$. Here, *d* is the average pore diameter, and $d_{1}$ the average pore diameter of sample $P_{1}$. In Figure 9, we show the variation of $\eta_{a}$ with $F^{\prime }$. We observe that the $F_{m}$ for the three sample collapse around $F^{\prime }=F^{*}\approx 0.055$. For $F^{\prime }<F^{*}$, $\eta_{a}$ decreases with $F^{\prime }$, while it increases with $F^{\prime }$ when $F^{\prime }>F^{*}$, suggesting the proposed blowing ratio $F^{\prime }$ is practically applicable in unifying the transpiration cooling characteristics of the sintered wire mesh in this study. We also note that the differences in $\eta_{a}$ among the three samples are insignificant when $F^{\prime }<F^{*}$, while the differences becomes significant when $F^{\prime }>F^{*}$. This can be interpreted from the combining effect of the internal cooling and external cooling. For the internal cooling, its heat transfer monotonically increases with the blowing ratio; for external cooling, its heat transfer first decreases and then increases with the blowing ratio. Therefore, on one hand, at low blowing ratio ($F^{\prime }<F^{*}$), these two cooling parts compensate each other, making the effect of geometry parameter insignificant. On the other hand, for large blowing ratio ($F^{\prime }>F^{*}$), these two cooling parts superimpose on each other, magnifying the effect of geometric parameter.

## 4. Conclusions

In summary, the transpiration cooling characteristics of the sintered wire mesh are systematically investigated and presented in this study. By measuring the surface temperature of the three test samples with different porosities at various blowing ratios, we observe the nonuniform distribution of surface temperature both along the flow direction and the transverse direction caused by the entrance effect and boundary layer in the corner, respectively. The transpiration cooling efficiency, which is determined by the internal forced convection and external film cooling, first decreases and then increases with the blowing ratio; however, it increases and then decreases with the porosity of the material. The former characteristic is caused by the external film cooling, while the latter is a combination effect from the internal and external cooling. We further propose a modified blowing ratio by the normalized pore diameter of the material to collapse the minima of *F* for all tested samples, which separates the decreasing and increase parts in the curve of transpiration cooling efficiency.

## Figures and Tables

**Table 1:** Geometric parameters of the tested sintered wire meshes.

No.	Diameter of Stainless Steel Wire (μm)	Quantity of Layers	Average Pore Diameter (μm)	Porosity *ε*
P1	140	40	165.5	56.5%
P2	140	42	107.8	46.6%
P3	140	54	71.8	38.9%

## Data Availability

All processed data in this study are included in this published article. Raw data will be provided on request from the corresponding author.
